# Bilateral otomastoiditis caused by *Cryptococcus neoformans*, a rare manifestation in patient with adult-onset immunodeficiency due to anti-interferon-γ autoantibodies

**DOI:** 10.1016/j.mmcr.2026.100808

**Published:** 2026-06-25

**Authors:** Punsita Tangkum, Ophat Janphet, Jakapat Vanichanan

**Affiliations:** a Division of Infectious Diseases, Department of Medicine, Faculty of Medicine, Chulalongkorn University, Bangkok, Thailand; b Department of Internal Medicine, Surin Hospital, Surin, Thailand

**Keywords:** Otomastoiditis, *Cryptococcus*, Anti–IFN-γ autoantibodies, Adult-onset immunodeficiency

## Abstract

*Cryptococcus* is an opportunistic fungal pathogen found in immunocompromised patient such as HIV or transplant recipients. Infection of central nervous system is the most common manifestation but pulmonary and skeletal involvement have been reported in non-HIV population. We report a case of cryptococcal otomastoiditis which was extremely rare and majority of cases were reported in individual without comorbidity. In this case, we also identified adult-onset immunodeficiency associated with interferon-gamma autoantibodies in this patient as an important underlying which predisposed cryptococcal infection.

## 1 Introduction

*Cryptococcus* is a pathogenic yeast belonging to the phylum Basidiomycota that causes invasive infections in immunocompromised individuals worldwide. This microorganism was initially recognized as a major etiologic agent of meningitis and meningoencephalitis among patients with acquired immunodeficiency syndrome (AIDS) following human immunodeficiency virus (HIV) infection [1]. In recent years, an increasing number of cryptococcal infections have been reported in non-HIV populations, including solid-organ transplant recipients, hematopoietic stem cell transplant recipients, and individuals receiving T-cell–depleting immunosuppressive therapy [1], [2].

Central nervous system (CNS) remains the most commonly affected organ system in both HIV and non-HIV populations. However, pulmonary, cutaneous, and skeletal infections appear to occur more frequently in non-HIV patients [3]. Otomastoiditis is a complicated infection involving the middle ear and mastoid air cells that may lead to permanent hearing loss if not appropriately treated. Bacterial pathogens are the most common cause of otomastoiditis, particularly *Streptococcus pneumoniae* and *Staphylococcus aureus* [4].

Fungal otomastoiditis is rare and typically occurs in immunocompromised individuals, with *Aspergillus* species reported as the most frequent causative pathogens [5]. Otomastoiditis caused by *Cryptococcus* is extremely uncommon. Interestingly, among reported patients, majority of them did not have established underlying comorbidity [[6], [7], [8], [9], [10], [11]]. Herein, we describe a case of bilateral cryptococcal otomastoiditis in an immunocompromised patient subsequently diagnosed with adult-onset immunodeficiency (AOID) associated with anti–interferon-gamma (IFN-γ) autoantibodies.

## 2 Case presentation

A 34-year-old Thai woman presented with progressive bilateral hearing loss. She had no known underlying medical conditions; however, three years earlier she had developed chronic cervical lymphadenopathy with granulomatous inflammation. Extensive investigations failed to identify a causative organism. Nevertheless, levofloxacin, clarithromycin, and trimethoprim–sulfamethoxazole (TMP/SMX) were empirically initiated for presumed non-tuberculous mycobacterial (NTM) infection. Antibiotics were discontinued after two years of treatment with complete resolution of lymphadenopathy.

Four months prior to presentation (day 0), the patient developed left-sided ear fullness followed by painless otorrhea. Two months later (day +58), she experienced worsening hearing loss accompanied by swelling of the left postauricular region. She visited a local hospital, where chronic otitis media was diagnosed and a four-week course of antibiotics was prescribed without improvement. One month before presentation (day +86), she noticed progressive hearing loss in both ears along with left-sided facial drooping. She was subsequently referred to our center (day +118) for further evaluation and management.

Physical examination revealed bilateral conductive hearing loss with granulation tissue obliterating both external auditory canals. Left-sided facial nerve palsy was observed, along with erythema, swelling, tenderness, and fluctuance over the left postauricular and temporal areas (Fig. 1). Computed tomography (CT) of the temporal bone demonstrated bilateral mastoid opacification with bony destruction, more extensive on the left side, suggestive of bilateral chronic otomastoiditis. Skull base osteomyelitis and right sigmoid sinus thrombosis were also identified (Fig. 2). However, neither bone scan nor positron emission tomography (PET) scan was performed.

**Fig. 1:**
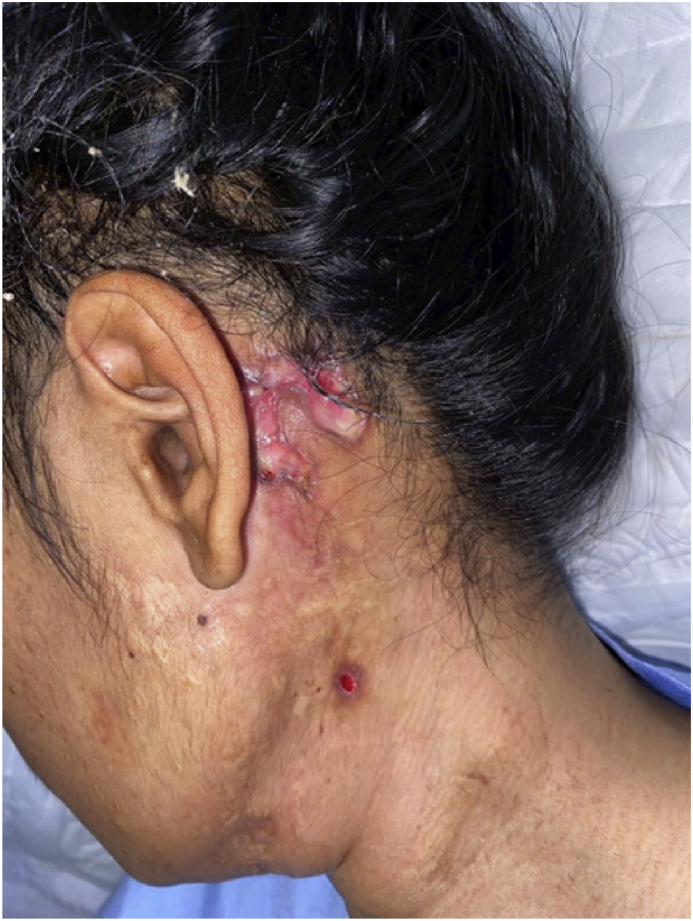
Swelling, redness with fluctuation at left postauricular extended to temporal area.

**Fig. 2:**
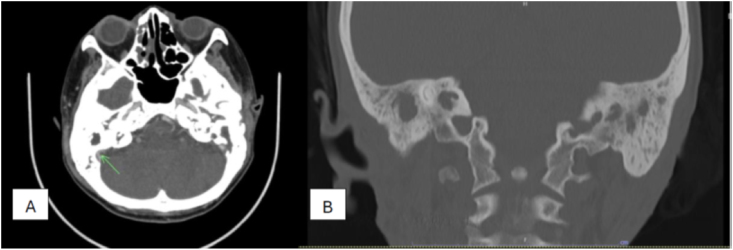
Computed tomography of the temporal bones. (A, axial view) Bilateral otomastoiditis, more extensive on the left with bony destruction with a filling defect in the left sigmoid sinus (arrow), consistent with cerebral venous sinus thrombosis. (B, coronal view) Bilateral mastoid opacification, more extensive on the left, with loss of bony septations and skull base.

Empirical intravenous vancomycin and piperacillin–tazobactam were initiated. The patient subsequently underwent bilateral mastoidectomy with abscess drainage (day +120). Intraoperative findings revealed extensive granulation tissue, purulent material, and necrotic bone in both mastoid cavities, along with pus collection in the left temporalis space. Gram stain, acid-fast bacilli staining, and modified acid-fast staining were negative. However, pus culture obtained from the left mastoid cavity grew *Cryptococcus neoformans*. Histopathological examination of mastoid tissue revealed encapsulated round-to-oval yeast with clear halos on hematoxylin and eosin (H&E) staining (Fig. 3A). Grocott–Gömöri methenamine silver (GMS) staining demonstrated narrow-based budding yeast (Fig. 3B), and mucicarmine staining was positive (Fig. 3C), confirming cryptococcal infection.

**Fig. 3:**
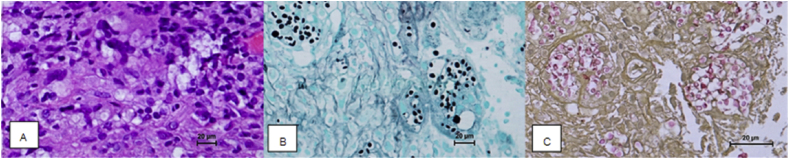
Histopathology of the mastoid lesion. (A–C) Yeast cells are identified as encapsulated round to oval organisms with clear halos on H&E (A), Positive budding yeast on Grocott's methenamine silver (B), and bright red–stained capsules on mucicarmine (C), confirming cryptococcal infection.

Cerebrospinal fluid (CSF) analysis showed a white blood cell count of 3 cells/mm^3^, red blood cell counts of 200 cells/mm^3^, glucose level of 52.3 mg/dL (serum glucose 95 mg/dL), and protein level of 28 mg/dL. India ink staining, cryptococcal antigen testing, and fungal culture from CSF were all negative. Serum cryptococcal antigen and HIV testing were also negative. Her absolute lymphocyte count was 540 cells/μl with CD4 count of 288 cells/mm^3^ (30%). Immunoglobulin G level was 1006 mg/dL. Further immunologic evaluation was performed which revealed positive anti– IFN-γ autoantibodies using enzyme-linked immunosorbent assay (ELISA) and impaired STAT1 phosphorylation. The test results were consistent with adult-onset immunodeficiency syndrome (AOID). ELISA for anti–granulocyte-macrophage colony-stimulating factor (GM-CSF) autoantibodies was also sent which yielded negative result.

Following surgery, the patient received induction therapy with amphotericin B deoxycholate (1 mg/kg/day) and flucytosine (100 mg/kg/day) for two weeks, followed by consolidation therapy with oral fluconazole (800 mg/day). Follow-up imaging demonstrated improvement of osteomyelitis with resolution of otorrhea, although hearing loss persisted.

## 3 Discussion

Otomastoiditis is an infection involving both the middle ear and mastoid air cells, typically occurring as a complication of otitis media. As the infection progresses, the mucosal lining of the mastoid air cells becomes inflamed and filled with exudative material. Prolonged infection may result in bone necrosis, destruction of the bony septa, and abscess formation [12].

Patients with otomastoiditis commonly present with hearing loss and malodorous otorrhea, whereas otalgia is reported in approximately 60% of cases [4]. The causative pathogens of acute otomastoiditis generally mirror those responsible for external and middle ear infections, including *Streptococcus pneumoniae*, *Staphylococcus aureus*, *Haemophilus influenzae*, and *Pseudomonas* species. In contrast, chronic otomastoiditis may be caused by unusual organisms such as mycobacteria and fungi [12]. Therefore, thorough microbiological investigation is essential in chronic cases, particularly in patients who fail to respond to broad-spectrum antibiotics.

Fungal otomastoiditis is rare and typically occurs in immunocompromised patients or in those with chronic ear disease who have received prolonged antibiotic therapy. However, it can occasionally occur in immunocompetent individuals and may lead to severe or life-threatening complications. *Aspergillus* species are the most frequently reported fungal pathogens, while other fungi such as *Candida*, *Blastomyces*, *Scedosporium apiospermum*, and Mucorales have also been described. The pathogenesis of fungal otomastoiditis is thought to involve direct extension from contiguous structures such as the middle ear or nasopharynx rather than hematogenous dissemination, which may explain the frequent unilateral presentation [4], [13].

*Cryptococcus* is a well-recognized opportunistic pathogen in immunocompromised individual. Bone and joint infections were reported more frequently in non-HIV patients [3]. Among cases with skeletal involvement, the thoracic bones are most commonly affected, followed by the vertebrae, upper extremities, and skull [14]. Skull osteomyelitis is thought to result either from hematogenous dissemination or extension from CNS infection. In the present case, bilateral cryptococcal otomastoiditis was confirmed by tissue culture and histopathology. This condition is extremely rare; according to our literature review, only 6 cases have previously been reported [[6], [7], [8], [9], [10], [11]]. Almost all patients developed temporal bone infection and otomastoiditis following cryptococcal meningitis, indicated meningogenic or hematogenous spreading as the main pathogenesis [[6], [7], [8], [9], [10]]. While another case was an immunocompetent patient without evidence of infection to other organ [11]. Finding in this case supported a possibility of tympanogenic route of infection. Similarly, our patient had negative cryptococcal antigen and fungal cultures from both blood and CSF, highlighting the diagnostic challenges of cryptococcosis in HIV-negative individuals.

Host immune responses against *Cryptococcus* rely primarily on type-1 T-helper (Th1) cell–mediated immunity. Therefore, individuals with impaired T-cell function, such as transplant recipients or those receiving immunosuppressive therapy, are particularly susceptible to invasive infection [15]. Adult-onset immunodeficiency associated with anti–IFN-γ autoantibodies has recently been increasingly recognized in previously healthy individuals presenting with disseminated infections caused by intracellular pathogens controlled by Th1 immunity. Pathogens commonly associated with this condition include non-tuberculous mycobacteria, *Salmonella, Cryptococcus*, and dimorphic fungi. Interestingly, many patients with disseminated fungal infections also have concurrent infections with other organisms, particularly NTM [16].

In our case, the patient had previously developed granulomatous cervical lymphadenitis that responded to treatment for presumed NTM infection. This history provided an important clinical clue prompting further evaluation for AOID associated with anti–IFN-γ autoantibodies. Currently *anti*-GM-CSF autoantibodies have been increasing recognized as an important underlying AOID leading to invasive cryptococcal infections which should be tested in previously healthy patients [17].

According to recent clinical guidelines for non-CNS disseminated cryptococcosis, induction therapy with amphotericin B combined with flucytosine for at least two weeks remains the standard treatment, followed by consolidation and maintenance therapy with fluconazole for a total duration of approximately 12 months [18]. Management of cryptococcosis in patients with AOID associated with anti–IFN-γ autoantibodies may require special considerations. The response rate to antimicrobial therapy alone has been reported to be less than 50%. Adequate source control is therefore essential, and prolonged antimicrobial therapy or adjunctive treatment with rituximab may be required to achieve favorable clinical outcomes [19].

In conclusion, we report a rare case of cryptococcal otomastoiditis in a patient without known immunosuppressive conditions at presentation. The diagnosis was challenging due to the absence of positive serologic biomarkers and negative CSF studies. This case highlights the importance of considering underlying adult-onset immunodeficiency associated with anti–IFN-γ autoantibodies in patients with unusual or disseminated fungal infections.

## Ethical form

Please note that this journal requires full disclosure of all sources of funding and potential conflicts of interest. The journal also requires a declaration that the author(s) have obtained written and signed consent to publish the case report /case series from the patient(s) or legal guardian(s).

The statements on funding, conflict of interest and consent need to be submitted via our Ethical Form that can be downloaded from the submission site www.ees.elsevier.com/mmcr. **Please note that your manuscript will not be considered for publication until the signed Ethical Form has been received.**

## Ethical statement

Written informed consent was obtained from the patient for publication of this case report and accompanying images. A copy of the written consent is available for review by the Editor-in-Chief of this journal on request.

## CRediT authorship contribution statement

**Punsita Tangkum:** Writing – original draft. **Ophat Janphet:** Supervision, Visualization. **Jakapat Vanichanan:** Conceptualization, Supervision, Writing – review & editing.

## Funding

There is no funding for this case report.

## Conflict of interest

All authors do not have conflict of interest.
